# The association between of placenta previa and congenital abnormalities: a systematic review and network meta-analysis

**DOI:** 10.1186/s12887-023-04433-z

**Published:** 2023-11-30

**Authors:** Ensiyeh Jenabi, Saeid Bashirian, Sahar Khoshravesh

**Affiliations:** 1 Autism Spectrum Disorders Research Center, Hamadan University of Medical Sciences, Hamadan, Iran; 2 Social Determinants of Health Research Center, Hamadan University of Medical Sciences, Hamadan, Iran; 3 Department of Community Health Nursing, School of Nursing and Midwifery, Hamadan University of Medical Sciences, Hamadan, Iran; 4 Chronic Diseases (Home Care) Research Center, Hamadan University of Medical Sciences, Hamadan, Iran

**Keywords:** Placenta previa, Congenital Abnormalities, Systematic review, Meta-analysis

## Abstract

**Background:**

Congenital abnormalities, as one of the fetal complications of placenta previa, may cause health problems or disability of the child throughout life. This study aimed to determine the relationship between placenta previa and congenital abnormalities.

**Methods:**

Potential articles were retrieved from three electronic databases (PubMed/Medline, Scopus, and Web of Sciences) up to 21 May 2023 without limit of time and language. A random effect model was applied for meta-analysis. The heterogeneity was calculated based on I^2^ statistic and Cochrane Q-test. All analyses were conducted at the significance level of 0.05 using STATA software, version 14. The quality assessment of the included studies was performed using the improved Newcastle-Ottawa Scale.

**Results:**

In the initial search, 829 articles were retrieved. Finally, according to the inclusion criteria, eight studies were analyzed in the meta-analysis. A significant association was reported between placenta previa and risk of congenital abnormalities based on crude form (OR = 1.81, 95% CI = 1.34 to 2.28) and adjusted studies (OR = 6.38, 95% CI = 1.47 to 11.30). The high heterogeneity was observed among the studies reported based on adjusted and crude form, respectively (I^2^ = 97.9%, P = 0.000) (I^2^ = 80.6%, P = 0.000). Therefore, publication bias was not observed among studies. Seven studies of the included studies were of high quality.

**Conclusion:**

Our study provides evidence that there is a positive and significant association between placenta previa and congenital malformations, including all structural anomalies, chromosomal defects, and congenital hypothyroidisms. Therefore, monitoring congenital abnormalities in the fetus of a mother with placenta previa is necessary.

**Supplementary Information:**

The online version contains supplementary material available at 10.1186/s12887-023-04433-z.

## Introduction

Placenta previa is the complete or partial coverage of the internal cervical os with the placenta [1]. It is an important cause of maternal and fetal morbidity and mortality in pregnant [2]. The overall prevalence of placenta previa is estimated at 5.2 per 1000 pregnancies [3]. The exact etiology of placenta previa is unknown; however, prior cesarean section, previous placenta previa, and abortions, smoking during pregnancy, termination of pregnancy, intrauterine surgery, multifetal gestation, multi-parity, assisted reproductive technology, and advanced maternal age can be risk factors for placenta previa [4–9]. The most accurate and safest diagnosis method of placenta previa is ultrasonography [10].

Placenta previa causes both maternal and fetal complications. Life-threatening maternal outcomes include hemorrhage, and shock [11, 12]. Evidence indicates that postpartum anemia, blood transfusion, hysterectomy, septicemia, thrombophlebitis, and delayed discharge from the hospital are other maternal complications of placenta previa [11–14]. Babies born to mothers with placenta praevia are more likely to suffer from Apgar scores < 7 at 1 and 5 min, small for gestational age, low birth weight, congenital abnormalities [14], require resuscitation, and neonatal intensive care unit (NICU) admission [15], stillbirth and neonatal death [11, 12].

Congenital abnormalities as one of the fetal complications of placenta previa, include all structural abnormalities (cardiovascular, digestive, respiratory, ear and nose, genital and urinary tracts, skin, musculoskeletal and nervous) and chromosomal abnormalities [12] which may cause health problems or disability of the child throughout life. In such a way that, based on the 2010 Global Burden of Disease (GBD) study [16, 17], congenital abnormalities cause 510 400 deaths in the world, 1% of all deaths, and rank 23rd among all causes of death [16]. In fact, deaths due to congenital abnormalities are really early and the burden in years of life lost (YLL) is higher [18]. Evidence indicates that although neonatal *complications* such as prematurity, stillbirth, and neonatal death *have been well-documented* [11, 12, 19, 20], *knowledge of the association of placenta previa with congenital* abnormalities *is limited* [21]. *It seems* that considering the importance of mortality and morbidity management of placenta previa in mother and fetus, especially the complication of congenital abnormalities, it is necessary to systematically study the relationship between placenta previa and congenital abnormalities. Our hypothesis in the present study was that there is a relationship between placenta previa and congenital abnormalities. These results may be able to provide appropriate solutions to the health system to better manage the burden of this problem. To the best of our knowledge, it was found that no systematic study has been done in this field. Therefore, the aim of this study was to determine the association between placenta previa and congenital abnormalities in the form of a systematic review.

## Methods

### Data sources

This study was performed based on the Preferred Reporting Items for Systematic Reviews and Meta-Analyses (PRISMA) guidelines [22]. Potential articles were retrieved from three electronic databases (PubMed/Medline, Scopus, Web of Sciences, science direct, and Embase) up to 21 May 2023 without limit of time and language. This systematic review as approved by the Research Ethics Committee of Hamadan University of Medical Sciences (No. IR.UMSHA.REC.1402.441).

### Search strategy

The search strategy was developed using Medical Subject Headings (MeSH). The search strategy was performed by keywords: (Placenta Previa or Placental Previa) and (fetal anomalies or fetal abnormality or congenital abnormalities or fetal malformation or congenital defects). The search strategy for PubMed/Medline is described in Supplementary file.

### Inclusion and exclusion criteria

Inclusion criteria were all full texts that identified the effect of placenta previa on congenital abnormalities. Qualitative studies, review studies, letters and correspondence, editorials, and conference proceedings were excluded.

### Data extraction

The results of initial searches were independently screened by two authors (EJ and SK) according to titles, abstracts, and full texts. Any disagreement among the researchers regarding the exclusion or inclusion of articles in the study was resolved with discussion. All searched articles in the initial search were entered into EndNote X8 software. The data in extraction form were; first author, publication year, study design, study period, study subjects, placenta previa diagnosis, congenital abnormalities type, total sample size (subject with placenta previa), crude OR (95% CI), adjusted OR (95% CI); adjustment factors, and quality of study. To minimize retrieval bias, the inclusion criteria were manually checked for additional eligible documents that could have been missed during the mentioned database and grey literature search.

### Heterogeneity assessment and publication bias

The heterogeneity was calculated based on I^2^ statistic [23] and Cochrane Q-test. The heterogeneity with I^2^ > 50% was high heterogeneity. For assessing publication bias, Egger’s [24] and Begg’s [25] tests were used.

### Statistical analysis

We applied a random effect model for meta-analysis. The effect size was calculated using the odds ratio (OR) with the 95% confidence interval (CI). All analyses were conducted using STATA 14 (StataCorp, College Station, TX, USA). Furthermore, for all statistical analyses, the significance level was set to 0.05.

#### Quality assessment tool

The quality assessment of the included studies was performed using the improved Newcastle-Ottawa Scale [26]. This scale has three sections including election (four items), comparability (two items), and exposure/outcomes (three items), and ranges from zero to nine. Two researchers conducted the quality assessment independently. Scores ≥ 7 points as high quality and scores < 7 points as low quality were considered. None of the studies were excluded based on quality assessment results.

## Results

### Results of the search and the included studies

**Fig. 1:**
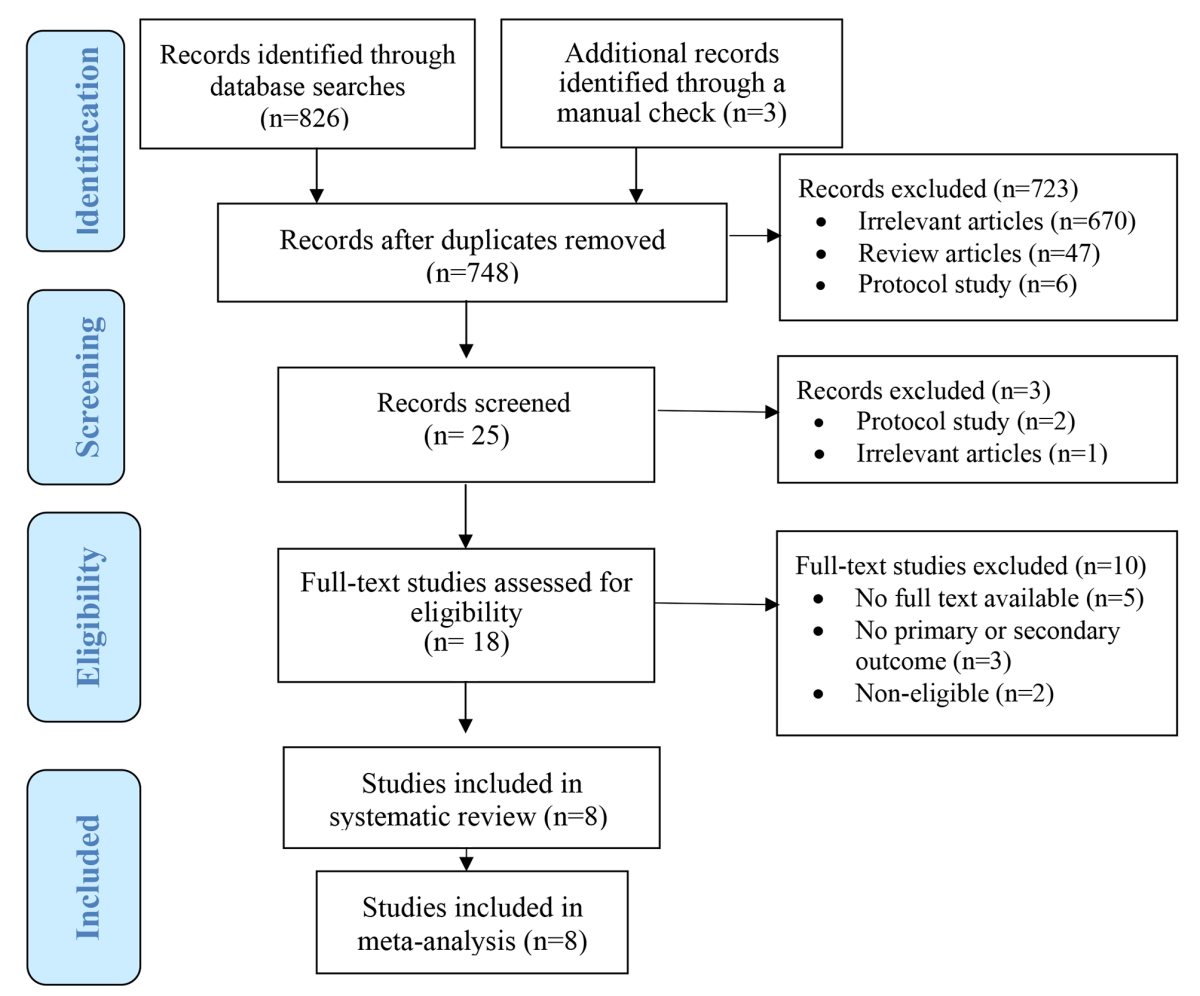
PRISMA flow diagram of the systematic review and meta-analysis selection process

A total of 829 articles were retrieved from the three electronic databases, PubMed/Medline, Scopus, Web of Sciences, science direct, and Embase. Three articles were identified by the manual check. After excluding 81 duplicate studies, we have identified articles by title, abstract, and full text. Finally, eight studies were reviewed in this systematic review (Fig. 1).

The included studies in this meta-analysis were: seven cohort studies and one case-control studies. Details of the included studies are presented in Table 1.

**Table 1:** Main characteristics of the included studies in the systematic review and meta analyses

Row	First author, publication year	Study design	Study period	Study subjects	Placenta Previa diagnosis	Congenital abnormalities type	Total Sample size (subject with PP)	Crude OR (95% CI)	Adjusted OR (95% CI); adjustment factors	Quality of study
1	Anwar, 2020	Hospital-based Cohort	2012-15	Group A: PP with & Without congenital malformation. Group B: Absence of PP with & Without congenital malformation	Second or third trimester ultrasonography	All structural anomalies, chromosomal defects, and congenital hypothyroidisms	90, 223 (1076)	Not reported	Not reported	High
2	Kancherla, 2015	Population-based Cohort	2000-10	All singletons born at or after 22 ± 0 weeks of gestation	Second or third trimester ultrasonography	Congenital abnormalities (including Major structural anomalies and chromosomal defects)	621,163 (1644)	1.65, (1.35,2.02)	1.55; (1.27,1.90) Maternal age, parity, fetal Sex, smoking, Socioeconomic status, chorionic villus biopsy, IVF, pre-existing Diabetes, depression, preeclampsia, and prior caesarean Section	High
3	Rosenberg, 2011	Hospital-based Cohort	1988–2009	Singletons	Second or third trimester ultrasonography	Congenital abnormalities (types not specified)	185,476 (771)	2.4 (1.9,3.0)	Not reported	High
4	Salihu, 2003	Population-based Cohort	1997	Singleton, Livebirths	Birth certificate	Congenital abnormalities (types not specified)	3,772,369 (9656)	1.11 (0.99,1.26)	Not reported	High
5	Sheiner, 2001	Hospital-based Cohort	1990–98	Singletons over 22 weeks of Gestation	Ultrasound and During delivery	Congenital abnormalities Including structural and Chromosomal anomalies	78,524 (298)	2.6 (1.5,4.2)	16.1 (13.4,19.3); Unknown	High
6	Crane, 1999	Population-based Cohort	1988-95	Singletons over 20 weeks of Gestation	Ultrasound /at Deliveries by Double-set or Caesarean Section	Cardiovascular, gastrointestinal, Respiratory, otolaryngology, Genitourinary, dermatologic, musculoskeletal, neurologic, and Chromosomal anomalies	92,983 (305)	2.52 (1.52, 4.17)	2.48 (1.50,4.11); Maternal age	High
7	Neri, 1989	Hospital-based Case-Control	1980-86	Unknown	Ultrasound	Congenital heart defects	164 (82)	Not reported	Not reported	Low
8	Brenner, 1978	Hospital-based Cohort	1962-69	Singletons At each gestational age	1^131^-albuminalbumin radioisotope technique	Congenital abnormalities Including structural &Chromosomal anomalies	31,070 (185)	Not reported	Not reported	High

Note: PP = Placenta Previa

### Participants

In six studies, the study subjects were singletons [12, 14, 21, 27–29], and in two studies it was unknown [30, 31].

### Study time and settings

Three studies were published in 2011 or later [21, 29, 31]. Other studies were published in 2003 or earlier [12, 14, 27–29]. Settings of the included studies were both hospital-based [14, 27, 29–31] and population-based [12, 21, 28].

### Design of the studies

Of the eight included studies, seven had cohort design [12, 14, 21, 27–29, 31] and there was one case-control study [30].

### Placenta previa diagnosis

Several diagnostic methods for placenta previa were mentioned in the included studies. Four studies used only ultrasound [21, 29–31]. In two studies, placenta previa diagnosis was conducted by ultrasound and during delivery [12, 14]. In the study of Brenner et al., placenta previa was diagnosed by 1^131^-albuminalbumin radioisotope technique [27]. In the study of Salihu et al., placenta previa was considered [28].

### Congenital Abnormalities type

The four studies considered all structural and chromosomal congenital abnormalities [12, 14, 21, 27, 31]. In the study of Neri et al., only congenital heart defects were considered [30]. In two studies, only congenital abnormalities were mentioned and their type was not specified [28, 29].

### Sample size of subject with placenta previa

In four studies, less than 500 [12, 14, 27, 30], in one study, less than 800 [29], and in three studies, more than 1000 placenta previa samples participated in the included studies [21, 28, 31].

### Main analysis

**Fig. 2:**
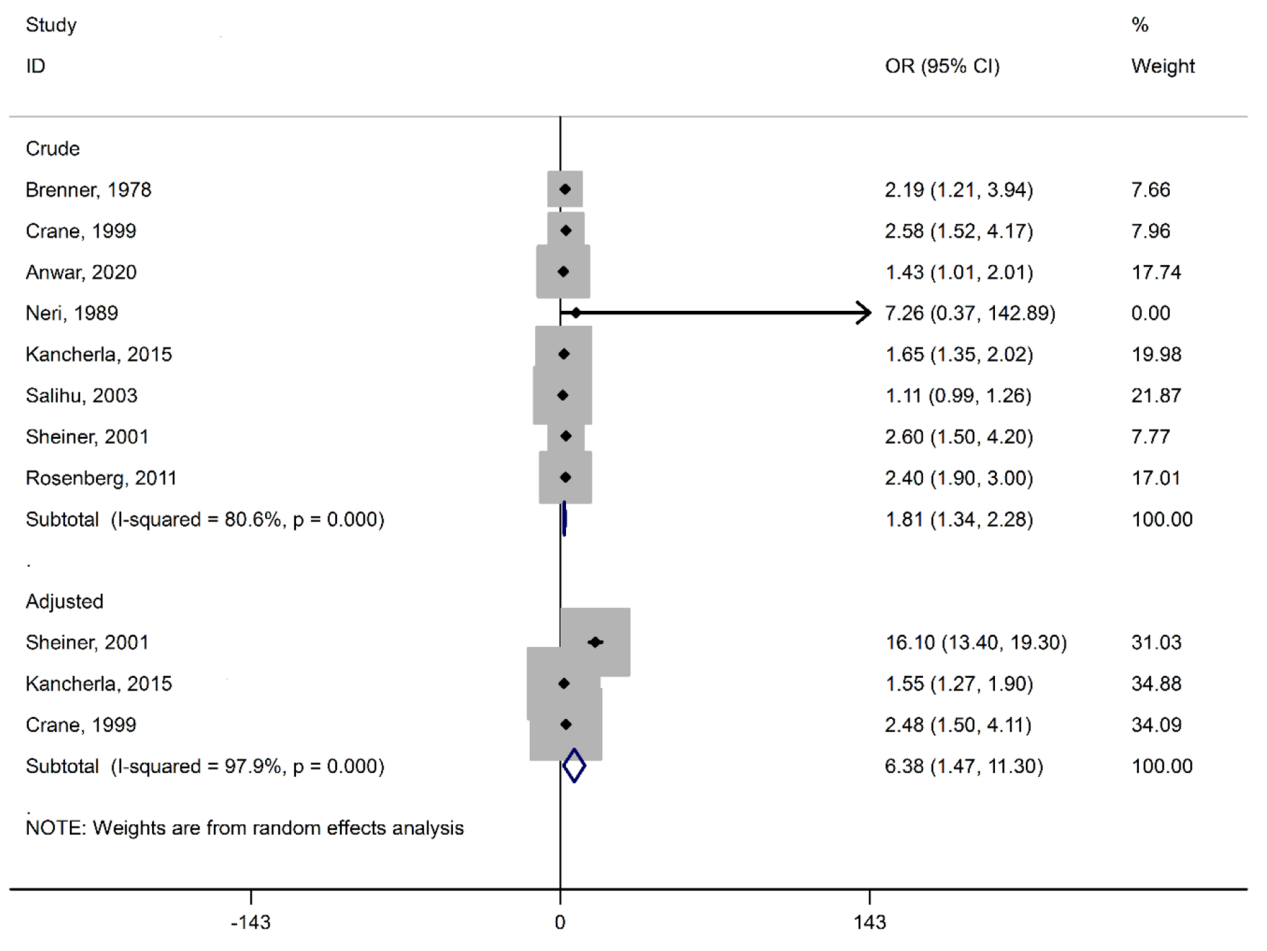
Forest plot of the association between ART and placenta previa based on OR

We presented the association between placenta previa and congenital abnormalities in Fig. 2 based on adjusted and crude variables. A significant association was reported between placenta previa and risk of congenital abnormalities based on crude form (OR = 1.81, 95% CI = 1.34 to 2.28) and adjusted studies (OR = 6.38, 95% CI = 1.47 to 11.30). The high heterogeneity was observed among the studies reported based on adjusted and crude form, respectively (I^2^ = 97.9%, P = 0.000) (I^2^ = 80.6%, P = 0.000). The P-values for Begg and Egger’s regression were 0.139 and 0.679. Therefore, publication bias was not observed among studies.

### Risk of Bias of the included studies

We did not exclude studies based on the results of the quality assessment. Seven studies of the included studies were high quality and one study was low quality according to the NOS Scale (Table 1).

## Discussion

To the best of our knowledge, this is the first meta-analysis that reports the association between placenta previa and congenital abnormalities. Our finding indicated that there was a significant association between placenta previa and risk of congenital abnormalities based on odds ratio reports in observational studies. In other words, the chance of congenital abnormalities of the fetus in mothers with placenta previa is 6.38 times higher than mothers without placenta previa. Evidence indicates that placenta previa can lead to adverse perinatal outcomes [4, 11, 32]. The effect of placenta previa on some adverse perinatal outcomes such as perinatal mortality and prematurity has been well-examined [19, 20], however, knowledge is still limited about its association with major congenital abnormalities [21]. Few population-based [12, 21, 28] and large hospital-based studies [14, 27, 29–31] have reported a positive association between placenta previa and congenital abnormalities but the association strength has widely varied due to differences in study designs, data sources, study subjects, sample size, selection criteria, method of placenta previa diagnosis, and lack of adequate information to control potential confounders [21, 31].

The pathophysiology of placenta previa is interesting due to its ability to cause congenital abnormalities. Based on embryology science, there is the highest risk of major congenital abnormalities in the 3–12 weeks after conception (critical window of organogenesis). In fact, a majority of birth defects are known to occur in the first 3 months of pregnancy [33]. Several probable mechanisms during organogenesis such as abnormal intra-uterine bleeding, hypoxia, and infection can be harmful to the fetus. In past studies, the positive association between placenta previa and intra-uterine infections and inflammation are reported [34, 35]. It is well-documented that placenta previa can cause bleeding throughout pregnancy [19]. In such a way that if the placenta remains in the lower and less vascularized region of the cervix for a long time, frequent contraction or cervical effacement and dilatation can lead to atrophy and asymptomatic bleeding, which could have damaging effects on the fetus [4]. Furthermore, the site of abnormal placental implantation can reduce the functional efficiency of the placenta [36] and cause fetal hypoxia [37]. The evidence shows that hypoxia during pregnancy lead to cardiovascular abnormalities [38]. It is well established that advancing maternal age and smoking are associated with genetic and chromosomal abnormalities. In women with advances maternal age and smoking, the higher incidence of antepartum complications such as miscarriage, gestational diabetes, placenta previa, and placental abruption have been approved. Therefore, chromosomal and genetic abnormalities may occur associated with placenta previa” [31].

A hospital-based study indicated that placenta previa was independently associated with a 16-fold increased risk of congenital abnormalities [14]. Also, Salihu et al., reported that mothers with placenta previa had a significantly higher chance of birthing infants with congenital abnormalities compared with mothers without placenta previa [28]. In 2015, a cohort study using a large population-based was conducted by Kancherla et al. in Finland [21]. They claimed that their study attempted to overcome the limitations reported in previous studies regarding the association between placenta previa and major congenital abnormalities [12, 14, 28–30]. Kancherla et al. found that there was a significant association between placenta previa and major congenital abnormalities in singleton births, however future studies are needed to investigate the association between placenta previa and individual types of congenital abnormalities [21]. In the conducted studies have pointed out the congenital abnormalities in two general types including structural and chromosomal abnormalities and it is not reported in detail. In the study of Anwar et al., in addition to structural and chromosomal abnormalities, hypothyroidism is also investigated [31]. It seems that it is necessary to conduct more studies regarding the relationship between placenta previa and congenital abnormalities type in order to determine which type of abnormality is more likely to occur in the fetus in the presence of placenta previa. Also, only in the study of Neri et al., the diagnosis method of abnormalities was specified. In such a way that congenital heart defects were diagnosed by complete cardiac workup which included physical examination, electrocardiography, chest roentgenogram and echocardiogram [30].

Confounding variables were controlled in some the included studies such as parity, fetal sex, smoking, socioeconomic status, chorionic villus biopsy, in vitro fertilization (IVF), pre-existing, diabetes, depression, preeclampsia, prior caesarean [21], and maternal age [12, 21]. As is clear, there is a lack of adequate information to control potential confounders in most of the included studies. Two strengths can be considered for the present study. First, seven out of eight studies were of high quality. Another strong point is that a significant relationship was found between placenta previa and congenital abnormalities.

Limitation.

High heterogeneity is one of the limitations of research, which even with controlling the confounding variables, may still remain high. Heterogeneity refers to diversity and differences among the population or samples under study. The presence of heterogeneity can make research results susceptible to influence and less generalizable to the population. Therefore, controlling confounding variables can be a method to reduce these types of effects. However, it is not always possible to control all confounding variables in research, and therefore high heterogeneity can be one of the limitations of research that can have negative effects on the generalizability of results.

## Conclusion

Our study provides evidence that there is a positive and significant association between placenta previa and congenital malformations, including all structural anomalies, chromosomal defects, and congenital hypothyroidisms. Therefore, monitoring congenital abnormalities in the fetus of a mother with placenta previa is necessary.

### Electronic supplementary material

Below is the link to the electronic supplementary material.

Supplementary Material 1

## Data Availability

All supporting data is available through the corresponding author.
