# Supplementary material for: The association between of placenta previa and congenital abnormalities: a systematic review and network meta-analysis
Source: BMC Pediatr. 2023 Nov 30;23:606. doi: 10.1186/s12887-023-04433-z (PMC10687781; doi:10.1186/s12887-023-04433-z)
Supplement: Supplementary file 1 — Supplementary Material 1 [file 12887_2023_4433_MOESM1_ESM.docx]

Appendix 1. The search strategy for PubMed/Medline

| "fetal anomalies"[Title/Abstract] OR "fetal abnormality"[Title/Abstract] OR "congenital abnormalities"[Title/Abstract] OR "fetal malformation"[Title/Abstract] OR "congenital defects"[Title/Abstract] OR “fetal defects” "[Title/Abstract] | [Congenital Abnormalities](https://www.ncbi.nlm.nih.gov/mesh/68000013) |
| --- | --- |
| "Placenta Previa"[Title/Abstract] OR "Placental Previa" [Title/Abstract] | Placenta Previa |
